# THE IMPACT OF NEGATIVE SOCIAL EXCHANGES ON ADULT PHYSICAL AND MENTAL HEALTH

**DOI:** 10.1093/geroni/igz038.519

**Published:** 2019-11-08

**Authors:** Abigail M Nehrkorn-Bailey, Julie Hicks Patrick, Madeline M Marello

**Affiliations:** West Virginia University, Morgantown, West Virginia, United States

## Abstract

As some health components may change across adulthood (CDC, 2019), social support for aging adults may be one way to optimize physical and mental health (U.S. Department of Health and Human Services, 2018). When social encounters are negative, however, physical and mental health may be negatively affected (Chen & Feeley, 2013; Hawkley & Cacioppo, 2010). Negative social exchanges (NSE) have been linked to an increase in negative affect and a decrease in positive affect (Newsom et al., 2003), along with an increase in physical symptoms (Edwards et al., 2001). In order to examine the relations between age, NSE, and two components of health (chronic health conditions and mental health) two moderated regression analyses were conducted using data from 848 adults (Mage = 32.5 years). Studying chronic health conditions, the overall model was significant, [F(3, 838) = 40.31, p < .001; R2 = .36]. Significant main effects emerged for NSE and age, along with a significant interaction between age and NSE (b = 0.010, p < 0.05). As NSE increased, the number of chronic health conditions increased, especially for older adults. For mental health, the overall model was significant [F(3, 845) = 52.96, p < .001; R2 = 0.16]. A significant main effect emerged for NSE, but neither the main effect for age nor the interaction were significant. Thus, although NSE can have deleterious effects on both mental and physical health, special attention needs to focus on the physical health of older adults who experience a higher number of NSE.

